# Active suppression of intestinal CD4^+^TCRαβ^+^ T-lymphocyte maturation during the postnatal period

**DOI:** 10.1038/ncomms8725

**Published:** 2015-07-21

**Authors:** Natalia Torow, Kai Yu, Kasra Hassani, Jenny Freitag, Olga Schulz, Marijana Basic, Anne Brennecke, Tim Sparwasser, Norbert Wagner, André Bleich, Matthias Lochner, Siegfried Weiss, Reinhold Förster, Oliver Pabst, Mathias W. Hornef

**Affiliations:** 1 Institute of Medical Microbiology and Hospital Epidemiology, Hannover Medical School, Carl-Neuberg-Straße 1, 30625 Hannover, Germany; 2 Institute of Medical Microbiology, RWTH University Hospital, Pauwelsstraße 30, 52074 Aachen, Germany; 3 Institute of Immunology, Hannover Medical School, Hannover, Carl-Neuberg-Straße 1, 30625 Hannover, Germany; 4 Institute of Infection Immunology, TWINCORE, Centre for Experimental and Clinical Infection Research, a joint venture between the Medical School, Hannover (MHH) and the Helmholtz Centre for Infection Research (HZI), Feodor-Lynen-Straße 7, 30625 Hannover, Germany; 5 Institute for Laboratory Animal Science, Hannover Medical School, Hannover, Carl-Neuberg-Straße 1, 30625 Hannover, Germany; 6 Department of Molecular Immunology, Helmholtz Centre for Infection Research, Inhoffenstraße 7, 38124 Braunschweig, Germany; 7 Department of Pediatrics, RWTH Aachen University Hospital, Pauwelsstraße 30, 52074 Aachen, Germany; 8 Institute of Molecular Medicine RWTH Aachen University Hospital, Pauwelsstraße 30, 52074 Aachen, Germany

## Abstract

Priming of the mucosal immune system during the postnatal period substantially influences host–microbial interaction and susceptibility to immune-mediated diseases in adult life. The underlying mechanisms are ill defined. Here we show that shortly after birth, CD4 T cells populate preformed lymphoid structures in the small intestine and quickly acquire a distinct transcriptional profile. T-cell recruitment is independent of microbial colonization and innate or adaptive immune stimulation but requires β7 integrin expression. Surprisingly, neonatal CD4 T cells remain immature throughout the postnatal period under homeostatic conditions but undergo maturation and gain effector function on barrier disruption. Maternal SIgA and regulatory T cells act in concert to prevent immune stimulation and maintain the immature phenotype of CD4 T cells in the postnatal intestine during homeostasis. Active suppression of CD4 T-cell maturation during the postnatal period might contribute to prevent auto-reactivity, sustain a broad TCR repertoire and establish life-long immune homeostasis.

## Abstract (web summary)

The mechanisms governing the ontogeny and maturation of the mucosal immune system during the postnatal period are not well understood. Here the authors characterize the homing kinetic, anatomical distribution and maturation of early intestinal CD4 T cells and provide insights into active T-cell suppression during the postnatal period.

## Graphical abstract

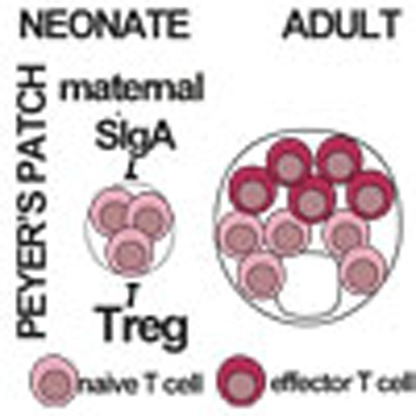

With birth, the sterile and protected mucosal body surfaces become exposed to environmental factors, nutritional constituents and the rapidly emerging microbiota. This process is paralleled by the maturation of the mucosal immune system. T lymphocytes generated in the thymus populate secondary lymphoid organs and generate a broad spectrum of adaptive T-cell immunity that ultimately plays a critical role in the establishment of life-long host–microbial homeostasis and antimicrobial host defence^1^,^2^,^3^,^4^. Interestingly, a fully developed intestinal mucosal immune system is present only comparably late after birth, long after microbial density reaches the level typically observed in adults^5^,^6^. Consistent with the hypothesis of a controlled inflammation, the large number of effector memory T cells in the homeostatic adult intestinal mucosa are thought to reflect the constant antigen exposure^7^. However, the precise kinetics of the cellular composition, the anatomical distribution and immune function during the early time window after birth has not been systematically investigated. Increasing evidence suggests that priming of the mucosal immune system during the postnatal period critically influences host susceptibility to certain immune-mediated diseases in later life. Epidemiological data suggest a relationship between the reduced prevalence of microbial infections and an increase of allergic and autoimmune diseases in humans^8^. Microbial exposure, composition of the enteric microbiota and infections during early childhood influence the development of allergies^9^,^10^,^11^,^12^. Consistently, in animal models, microbial immune stimulation during early development has been shown to influence immune cell recruitment and function as well as the susceptibility to allergic and autoimmune disease^13^,^14^,^15^,^16^,^17^,^18^. These findings have led to the definition of a ‘window of opportunity' during the early postnatal development, with life-long consequences for the host's immune system.

A detailed characterization of immune maturation during early childhood is a prerequisite for a comprehensive understanding of host–microbial homeostasis and disease susceptibility. We therefore characterized homing, composition and transcriptional profile of mucosal T cells during the early postnatal period and determined the kinetic of immune maturation. Surprisingly, CD4 T lymphocytes showed an immature phenotype during the homeostatic postnatal period despite the rapid establishment of the neonatal gut microbiota. Our results identify the active suppression of CD4 T-lymphocyte maturation and characterize the underlying mechanisms. These mechanisms might contribute to restrict the emergence of cross-reactive immune cells and sustain a broad TCR repertoire throughout infancy.

## Results

### The intestinal immune cell composition

**Figure 1:**
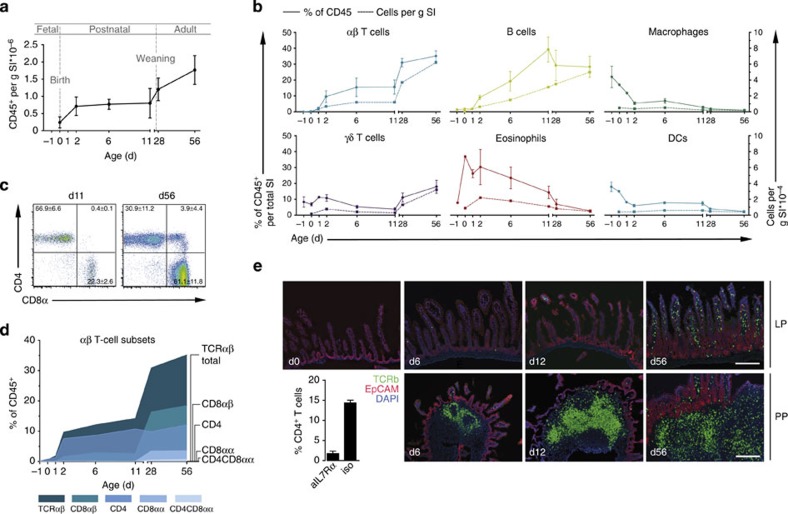
Kinetic of immune cell composition in the neonatal small intestine. (**a**) Immune cells were isolated from whole small intestinal tissue (SI) at the indicated age and absolute numbers of CD45^+^ cells were determined by FACS analysis normalizing to the simultaneously counted Trucount beads. Total recovered CD45^+^ immune cell numbers were divided by the average weight of the total organ at the indicated age and are presented as CD45^+^ cells per g tissue (mean±s.d.). (**b**) Major immune cell subsets were determined over time and are presented as percentage of viable CD45^+^ cells (solid line, left *y* axis) and absolute cell numbers per g tissue (broken line, right *y* axis). Cell subsets were defined by the following markers: TCRβ^+^, αβ T lymphocytes; TCRδ^+^, γδ T lymphocytes; CD19^+^, B lymphocytes; SiglecF^+^SSC^hi^, eosinophils; MHCII^+^CD11b^+^F4/80^+^, Macrophages; MHCII^+^CD11c^+^, DC. (**c**) αβ T lymphocyte subset analysis represented as FACS plots (CD4 and CD8α) from total small intestine of 11- and 56-day-old mice (*n*=6, mean±s.d., two experiments) and (**d**) as an age kinetic presented as percentage of viable CD45^+^ cells. Total TCRβ^+^ (dark blue), TCRβ^+^CD8αβ^+^ (turquoise), TCRβ^+^CD4^+^ (blue), TCRβ^+^CD8αα^+^ (light blue) and TCRβ^+^CD4^+^CD8αα^+^ (grey blue) subsets are represented. For all time points in **a**,**b**,**d**, *n*=4–11 pooled from three to five independent experiments. (**e**) Immunofluorescence staining of TCRβ (green) on SI tissue sections at the indicated ages. Images in the upper row are representative for the LP; images in the lower row illustrate PPs dissected using the binocular. Magnification, × 100; counterstaining with EpCAM (red) and DAPI (blue) (scale bar 100 μm). Bar graph: PPs were depleted by injection of the anti-IL7Rα anti body in pregnant dams at E14.5 gestation. Small intestinal CD4^+^ T lymphocytes were analysed in 11-day-old neonates from dams that received an anti-IL7Rα antibody (aIL7Ra) or an isotype control antibody (iso). (*n*=1 litter, mean±s.d.). d, days.

The emergence of immune cells in the murine neonate intestine has not yet been systematically characterized. We therefore performed a detailed analysis of the immune cell composition in total small intestinal tissue at various time points after birth. The number of CD45^+^ immune cells dramatically increased during the first 2 days after birth. Thereafter, a stable number of cells was maintained until day 11 with further increase only after weaning (Fig. 1a). Immune cell subset analysis revealed that myeloid cells (macrophages, dendritic cells (DCs) and eosinophils) and γδ T lymphocytes were present at birth and their numbers remained stable during postnatal development (Fig. 1b). In contrast, the kinetics of B and TCRαβ^+^ T-lymphocyte numbers largely mirrored the development of total CD45^+^ cell numbers. Thus, massive recruitment of B and TCRαβ^+^ T lymphocytes takes place during the first 2 days after birth (Fig. 1b). B-cell frequencies increased subsequently until a maximum at 3 weeks after birth in accordance with a recent report on B-cell recruitment and local generation^6^. In contrast, the number of TCRαβ^+^ T lymphocytes remained stable with respect to tissue weight throughout the postnatal period. A further linear increase in TCRαβ^+^ cell numbers occurred at the time of weaning. This increase was mainly due to the strong expansion of CD8αβ^+^TCRαβ^+^ T lymphocytes, which accounted for the majority of TCRαβ^+^ cells in the adult animal, but was also supported by the emergence of CD8αα^+^TCRαβ^+^ and CD4^+^CD8αα^+^TCRαβ^+^ T cells (Fig. 1c,d). The majority (66.7±6.6%) of pre-weaning TCRαβ^+^ T lymphocytes consisted of CD4^+^ cells and this cell population did not expand further after weaning.

Immunostaining of intestinal tissue sections of pre-weaning mice showed TCRαβ^+^ T lymphocytes to be localized almost exclusively in lymphoid aggregates (Fig. 1e). Since isolated lymphoid follicles are not yet present at birth, these aggregates represent nascent Peyer's patches (PP)^19^. In accordance, PP depletion by administration of anti-interleukin (IL)-7R antibodies^20^ largely abolished T cell accumulation in the early postnatal period (Fig. 1e, bar graph). Significant recruitment of TCR αβ^+^ T lymphocytes into the lamina propria (LP) was detected between days 11 and 28 only. The rapid recruitment after birth and the predominance and compartmentalized localization of CD4^+^TCRαβ^+^ T cells in the neonate mucosa represent a unique situation that is not observed in the adult host. We therefore focused on phenotypic and functional aspects of this cell population during the neonatal period.

### Phenotype of neonatal CD4^+^ T lymphocytes

**Figure 2:**
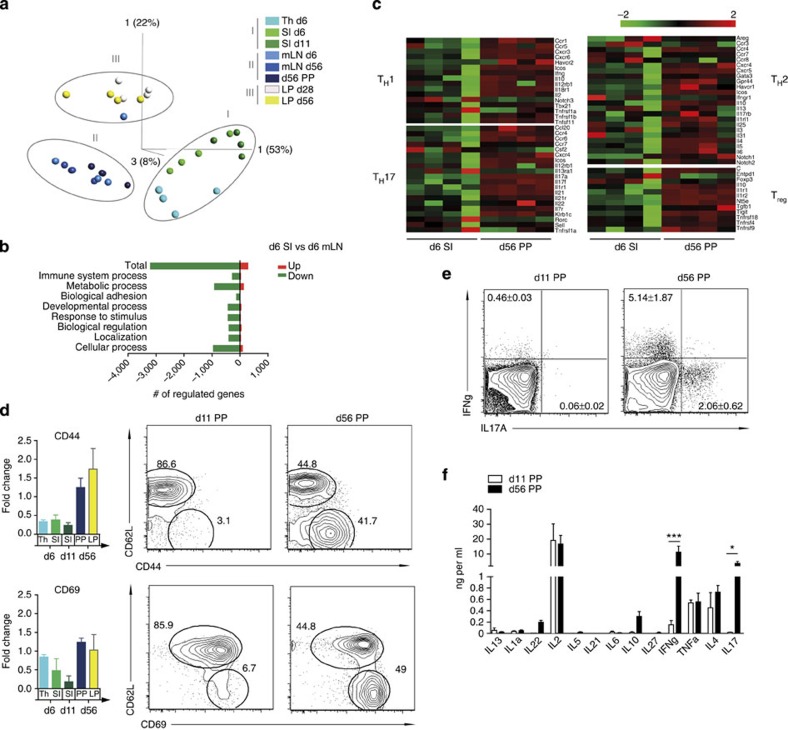
Transcriptional profiling and functional characterization of neonatal mucosal CD4 T lymphocytes. Transcriptome analysis of flow cytometry-sorted CD4^+^CD8^−^TCRβ^+^ cells isolated from the indicated organs/age of the animals (days, d). The array was performed in quadruplicates with each group comprising sorted cell populations pooled from one litter of neonate animals (d6 and day 11) and two to three adult mice per sample. Thymus (Th) d6, SI d6, SI d11, mLN d6, mLN d56, LP d28, LP d56 and PP d56. (**a**) Principal coordinate analysis of the groups based on 10,000 differentially regulated individual genes using multigroup comparison (ANOVA analysis) and a false discovery rate (FDR) of <10^4^. (**b**) Functional analysis of up- (red) and downregulated (green) genes of d6 SI compared with d6 mLN using two group analysis, FDR=0.05 and a fold-change cutoff of 2. Analysis was performed in PANTHER using the gene ontology term biological process. (**c**) Transcriptome analysis of signature T-helper genes d6 SI and d56 PP. (**d**) Fold-change values (normalized to the mean intensity of all samples) of CD44 and CD69 mRNA measured by microarray analysis in CD4 T lymphocytes from thymus (Th) and small intestine (SI) of 6-day-old mice, SI of 11-day-old mice and PPs and LP of 56-day-old mice (left panels) and comparative FACS analysis of the CD44 or CD69 versus CD62L surface expression on CD4 T lymphocytes in PP of 11-day-old mice and PP and LP of 56-day-old mice (right panels; *n*=4 for left and right panels, mean±s.d.). (**e**) Intracellular FACS staining for IFNγ and IL-17A in CD4 T cells isolated from PPs from 11- and 56-day-old mice restimulated with PMA/Ionomycin (*n*=4, representative of three experiments, mean±s.d.). (**f**) Cytokine detection by Multiplex Bead Array analysis in the supernatant of FACS-sorted CD4 T cells isolated from PPs of 11- and 56-day-old mice and restimulated with PMA/Ionomycin. (*n*=4, representative of two experiments, mean±s.d.; two-way ANOVA, Bonferroni's post test, ***P<0.001; *P<0.05).

CD4^+^ T lymphocytes in the adult LP display an exclusively mature phenotype and adult PPs contain a large proportion of effector memory type CD4^+^ T lymphocytes. To characterize the phenotype of postnatal intestinal CD4^+^ T cells and define anatomical site- and age-specific differences, we compared the transcriptome of CD4^+^ T cells from thymus (Th), total intestinal tissue (SI) at days 6 and 11 after birth, PPs of 56-day-old as well as LP of 28- and 56-day-old mice as well as mesenteric lymph nodes (MLN) at days 6 and 56 after birth. We identified a large number of significantly differentially expressed genes (*P*<0.001 by ANOVA (analysis of variance; Supplementary Fig. 1a). Expression profiles segregated in three clusters including (I) thymus and neonatal small intestinal cells (d6 Th, d6 SI, d11 SI), (II) neonatal and adult MLN cells and adult PP cells (d56 MLN, d6 MLN, d56 PP) or (III) adult LP cells (d28 LP, d56 LP; Fig. 2a). Thus, the gene expression profile of CD4^+^ T cells in d6 and d11 SI and that in d6 mLN diverged from CD4 single positive thymocytes into different directions, suggesting rapid acquisition of distinct functional phenotypes. Further gene expression analysis of neonatal intestinal CD4^+^ T lymphocytes (d6 SI), in contrast to their MLN counterparts (d6 mLN), suggested a functionally inactive state with a large number of significantly downregulated genes (Fig. 2b).

Consistent with this finding, transcriptome analysis revealed the absence of CD4^+^ effector memory cells in neonate small intestinal tissue illustrated by a lack of genes associated with T-cell polarization (Fig. 2c) as well as low mRNA expression of CD44 and CD69 (Fig. 2d, left panels). Low surface protein expression of CD44 and CD69 in combination with high CD62L expression on neonatal intestinal CD4^+^ T cells was confirmed by flow cytometry (Fig. 2d, right panels). IL-17A and interferon-γ (IFN-γ) are highly expressed in mucosal immune cells and have been shown to play a critical role in mucosal homeostasis and antimicrobial host defense in adult individuals^21^,^22^. Intracellular cytokine staining revealed strongly reduced synthesis of IL-17A and IFN-γ in neonatal as compared with adult CD4^+^ T cells (Fig. 2e). Consistently, cytokine secretion profiling revealed that also the secretion of IFN-γ and IL-17 was significantly reduced and the release of IL-10 and IL-22 was virtually absent after the restimulation of neonatal CD4^+^ T lymphocytes, whereas adult lymphocytes readily produced substantial amounts of these cytokines (Fig. 2f). Of note, the immature phenotype was not restricted to neonatal CD4^+^ T lymphocytes. Also neonatal CD8^+^ T lymphocytes exhibited low CD44 and high CD62L surface staining (Supplementary Fig. 1b).

Between days 11 and 28 after birth, intestinal CD4^+^ T cells start to populate the LP (Fig. 1e). Since adult LP CD4^+^ T cells almost exclusively exhibit an effector memory phenotype with high CD44 expression, we next determined the maturation status specifically of neonatal CD4^+^ T cells within the LP. Therefore, PPs were mechanically removed from the small intestinal tissue of 11-day-old neonates and the CD44 expression of the ‘LP enriched' fraction of CD4^+^ T cells was analysed (Supplementary Fig. 1c). Similar to the phenotype of total intestinal CD4^+^ T cells, the expression of CD44 on neonatal LP T cells was low providing further evidence for the sustained presence of naïve, immature CD4^+^ T lymphocytes in the neonate intestine throughout the postnatal period.

Importantly, CD4^+^ T cells in the neonatal intestine exhibited a sustained immature phenotype despite rapid colonization with enteric microbiota reaching density levels comparable to adults shortly after birth^23^,^24^. In contrast, CD62L^lo^CD44^hi^CD4^+^ T cells appeared in splenic tissue already at day 11 after birth (Supplementary Fig. 1d) despite a delayed recruitment of CD4^+^ T cells into the spleen compared with gut and a presumably lower contact with microbial antigens (Supplementary Fig. 1e). These results suggest that active suppressive mechanisms exist in the neonatal intestine that prevent maturation of CD4^+^ T lymphocytes.

### Origin and recruitment of CD4^+^ T lymphocytes

We next aimed at elucidating the origin and recruitment mechanism of neonatal small intestinal CD4^+^ T cells. In addition to release from the thymus, translocation of breast milk-derived maternal T lymphocytes has been described in piglets, lambs and calves and suggested to impact on neonatal immunity^25^,^26^. Maternal T cells might thus contribute to the early T-cell population in the neonate intestine. We employed the congenic marker genetic model (Ly5.1 and Ly5.2 parental animals) to investigate the presence of maternal lymphocytes within the murine neonate intestine. However, we did not detect (Ly5.1 or Ly5.2) single positive CD4^+^ T lymphocytes in offsprings of Ly5.1 × Ly5.2 crosses (Supplementary Fig. 2a). Alternatively, T lymphocytes might be generated in the neonatal intestine in analogy to the recently described local formation of B cells^6^. Local T-lymphocyte proliferation might further enhance their ability to efficiently populate the regional niches. To exclude local T-lymphocyte generation, *Rag-Gfp* mice were used. In these mice, *Rag1* induction during early lymphocyte development temporarily switches on green fluorescent protein (GFP) expression. The GFP protein level in lymphocytes subsequently declines^27^. CD4^+^ T lymphocytes in neonatal *Rag-Gfp* mice expressed GFP at intermediate levels indicating the absence of locally generated T lymphocytes (Supplementary Fig. 2b). In addition, mRNA expression levels of the cell division markers Ki67 and PCNA as well as BrdU incorporation as a marker for DNA replication were similar in neonate CD4^+^ T lymphocytes and adult controls (Supplementary Fig. 2c,d). We therefore believe that the recruitment of CD4^+^ T cells from the thymus fuels accumulation of T cells in the gut after birth.

**Figure 3:**
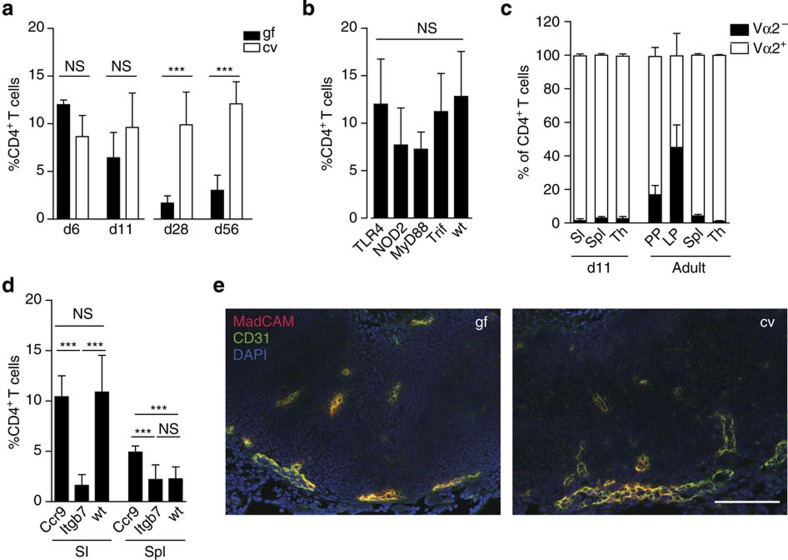
Postnatal homing of CD4 T cells to the neonatal intestine. (**a**) Comparative analysis of the percentages of CD4^+^TCRβ^+^ T lymphocytes among total CD45^+^ immune cells in intestinal tissue of conventional (cv) and germ-free (gf) mice at the indicated age (*n*=8–11 from two experiments, mean±s.d.; one-way ANOVA, Bonferroni's post test, ***P<0.001; NS, not significant). (**b**) Comparative analysis of the percentage of CD4^+^TCRβ^+^ T lymphocytes among total CD45^+^ immune cells in wild type (wt), Tlr4^−/−^, Trif^Lps2/Lps2^, MyD88^−/−^ and Nod2^−/−^ mice at 6 days after birth (*n*=4–11, mean±s.d.; one-way ANOVA, Bonferroni's post test; NS, not significant). (**c**) Comparative analysis of the frequency of Vα2^+^ and Vα2^−^ cells among TCRβ^+^CD4^+^ lymphocytes in the total small intestine (SI), spleen (Spl) and thymus (Th) of 12-day-old and PPs, LP, spleen (Spl) and thymus (Th) of adult OTII transgene mice (*n*=7 and 3, respectively; representative of two independent experiments, mean±s.d.). (**d**) Comparative analysis of the percentage of CD4^+^TCRβ^+^ T lymphocytes among total CD45^+^ immune cells in the small intestine (SI) and Spleen (Spl) of wild type (wt), Ccr9^−/−^ and Itgb7^−/−^ mice at 6 days after birth (*n*=7–11 from two experiments, mean±s.d.; one-way ANOVA, Bonferroni's post test, ***P<0.001; NS, not significant). Note that the same data set from the neonatal wt/cv group is shown in a–c because the figures represent pooled data. (**e**) Immunofluorescence staining of MadCAM-1 (red) in combination with CD31 (green) on PP tissue sections of gf and cv 11-day-old neonates dissected using the binocular. Magnification, 1 × 200; counterstaining with DAPI (blue; scale bar, 100 μm; *n*=1, representative of two experiments).

Since homing of CD4^+^ T cells from the thymus to the neonate intestine accompanies postnatal development of the enteric microbiota, we next assessed the influence of bacterial colonization and innate immune recognition. Surprisingly, postnatal recruitment of CD4^+^ T cells at 6 and 11 days after birth occurred irrespective of microbial exposure to a similar degree in germ-free and conventional animals, whereas the cell number was significantly reduced in the absence of the microbiota in adult animals (Fig. 3a and Supplementary Fig. 2e). Consistently, the analysis of mice deficient in the expression of innate immune receptors known to play a critical role in microbial recognition at the intestinal mucosa such as Nucleotide-binding oligomerization domain-containing protein 2 (Nod2), Toll-like receptor (Tlr)4, the Tlr adaptor molecule myeloid differentiation primary response gene 88 (MyD88) as well as the TIR-domain-containing adapter-inducing interferon-β (Trif) revealed no significant influence of innate immune stimulation on early CD4^+^ T-cell recruitment (Fig. 3b and Supplementary Fig. 2e)^28^,^29^,^30^.

In addition, we investigated the influence of TCR-mediated signalling, that is, the requirement of TCR specificity for T-cell homing. We therefore used OTII TCR transgenic mice on a Rag-sufficient genetic background. In addition to TCR transgenic OTII cells, these mice produce non-transgenic T cells with an oligoclonal repertoire of endogenously rearranged TCR loci. Similar studies in adult animals have shown that these Vα2Vβ5^−^ non-OTII T cells are enriched in PPs suggesting antigen-dependent CD4^+^ T-cell maintenance after weaning^31^. Here we compared the relative proportion of Vα2^−^ versus Vα2^+^ CD4^+^ T cells (non-OTII versus OTII cells, respectively) in the neonatal thymus, small intestine and spleen tissue at day 11 after birth. In contrast to the situation in adult mice, however, no enrichment of Vα2^−^ expression was found in small intestinal CD4^+^ T cells suggesting antigen-independent accumulation of mucosal CD4^+^ T cells during the postnatal period (Fig. 3c). This finding is also in accordance with the above-described innate immune receptor-independent CD4^+^ T-cell homing in the neonate host since innate immune sensing is a prerequisite for TCR activation.

Next, we analysed the role of the gut-homing molecules β7 integrin and CCR9 for CD4^+^ T-cell recruitment in the neonate intestine. The percentage and absolute number of TCRαβ^+^ cells in 6-day-old *Ccr9*-deficient mice was similar to wt, whereas *Itgb7-*deficient mice exhibited significantly reduced T-cell numbers in the small intestine compared with wt (Fig. 3d and Supplementary Fig. 2f). These findings are consistent with the expression of β7 integrin on neonatal intestinal CD4^+^ T lymphocytes (Supplementary Fig. 2g) and low mRNA expression of the CCR9 ligand CCL25 by epithelial cells of the neonate as compared with the adult intestine (array data accessible through GEO GSE35596 and GSE35597 (ref. 32)). To investigate β7 integrin-dependent but microbiota-independent homing of neonatal CD4^+^ T lymphocytes, expression of the β7 integrin ligand mucosal vascular addressin cell adhesion molecule 1 (MadCAM-1) on the intestinal vascular endothelium was investigated by immunostaining in PPs of GF and conventional neonates. As expected, intestinal CD31^+^ endothelial cells in the neonate intestine stained MadCAM-1 positive in both GF and conventional mice providing the retention signal for CD4^+^ T lymphocytes (Fig. 3e). In sum, these observations suggest that CD4^+^ T-cell recruitment to the small intestine during the postnatal period in mice occurs independent of microbial exposure, innate immune stimulation and TCR signalling but is based on β7 integrin-dependent pathways. This finding is fully consistent with the immature phenotype of T lymphocytes within the neonate intestine.

### Maturation and effector function

**Figure 4:**
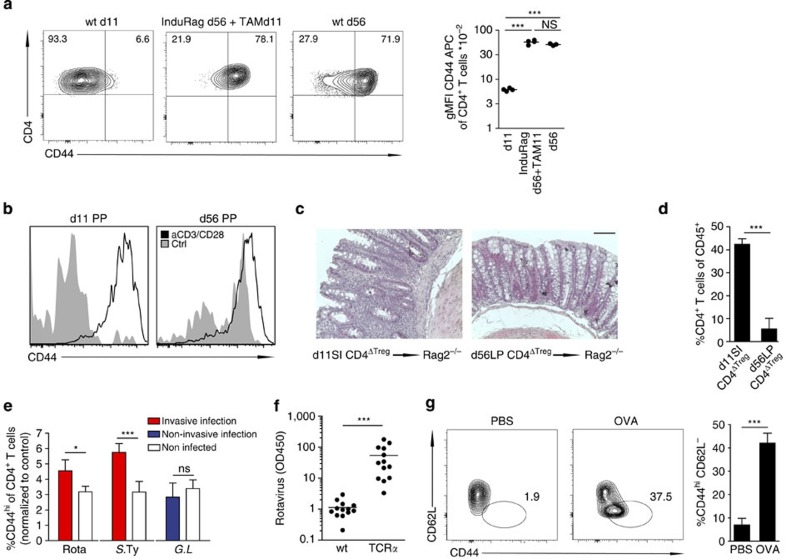
Maturation of neonatal CD4 T lymphocytes following mucosal challenge. (**a**) Lymphocyte maturation status in the adult lymphopenic host after the induction of Rag in InduRag mice. FACS analysis (left panel) and gMFI of CD44 (right panel) on CD4^+^TCRβ^+^ lymphocytes obtained from the SI of 11- and 56-day-old wt (wt d11 and wt d56) and adult InduRag mice 11 days after Tamoxifen administration (InduRag d56+TAM11). (*n*=3–4, representative of two experiments, mean; one-way ANOVA, Bonferroni's post test, ***P<0.001; NS, not significant). (**b**) *Ex vivo* activation of CD4^+^TCRβ^+^ lymphocytes from PP of 11- and 56-day-old mice after 3 days of culture in presence of anti-CD3/CD28 beads (*n*=4, one experiment). (**c**) H&E staining and (**d**) percentage of infiltrating CD4^+^TCRβ^+^ lymphocytes of CD45^+^ in the colonic tissue of adult Rag2 recipients 5–6 weeks after transfer of 1–5 × 10^4^ SI CD4^+^ T cells from 11-day-old or adult Foxp3-GFP donor mice after T_Reg_ depletion (T-cell transfer colitis model). Magnification, × 40 (bar graph 100 μm). (*n*=3–4, representative of two experiments, mean±s.d.; unpaired Student's *t*-test, ***P<0.001). (**e**) Percentage of CD44^hi^ cells among CD4^+^TCRβ^+^ lymphocytes (using the 3% of CD4 T cells in the non-infected control mice with the highest CD44 expression as a reference gate) in the neonate SI after infection with rotavirus (infected at d4 post parturition, pp and analysed at 8 d.p.i.), *S.* Typhimurium (infected at d4 pp and analysed at 4 d.p.i.) and *Giardia lamblia* (infected d4 pp and analysed at 8 d.p.i.). (One litter (*n*=6–11) from the infected group and four age-matched non-infected controls were analysed per experiment; representative of two experiments, mean±s.d.; unpaired Student's *t*-test, *P<0.05, ***P<0.001; NS, not significant). (**f**) Quantification of rotavirus antigen by ELISA in colon homogenate of wt and TCRα^−/−^ neonates at 8 d.p.i. infected at 4d pp (*n*=12–13 from two experiments, mean; Mann–Whitney *U*-test, ***P<0.001). (**g**) FACS plots (left panel) and percentages (right panel) of CD44^hi^CD62L^−^ of CD4^+^TCRβ^+^ lymphocytes in PPs. 11-day-old DO11.10 neonates were gavaged daily with 10 mg OVA or PBS starting at d3 pp (*n*=5, representative of two experiments, mean±s.d.; unpaired Student's *t*-test, ***P<0.001).

To investigate whether the sustained immaturity of thymus-derived lymphocytes is an intrinsic feature of T lymphocytes recently released from the thymus or a consequence of the local milieu in the neonate intestine, we analysed InduRag mice carrying an inducible *Rag1* gene^33^. Untreated InduRag mice are devoid of peripheral T cells due to the absence of Rag1 expression. Administration of Tamoxifen to adult InduRag mice induces the expression of Rag1 and generates a situation similar to the postnatal period with release of immature T cells from the thymus. Intestinal CD4^+^ T cells in adult conventional InduRag mice at 11 days after Tamoxifen administration had acquired similarly elevated CD44 expression levels as observed in adult wt animals (Fig. 4a). Thus, sustained immaturity of CD4^+^ T cells is not an intrinsic consequence of their recent release from the thymus or accumulation of effector memory type cells with age but relies on age-dependent mechanisms within the local milieu.

To investigate the potential of neonatal CD4^+^ T cells to undergo maturation, we next tested the ability of neonatal CD4^+^ T cells to become activated and to acquire an effector memory phenotype *in vitro*. Incubation of CD4^+^ T cells obtained from the small intestine of 11-day-old neonates in the presence of anti-CD3/anti-CD28 coated beads for 3 days induced CD44 expression and proliferation to a similar degree as incubation of CD4^+^ T cells isolated from adult PPs (Fig. 4b and Supplementary Fig. 3a). In addition, neonatal CD4^+^ T lymphocytes were tested *in vivo*. CD4^+^ T lymphocytes obtained from 11-day-old neonates were compared with adult CD4^+^ T lymphocytes for their ability to mature and acquire effector function in an adoptive T-cell transfer colitis model. Transfer of neonatal intestinal CD4^+^ T cells depleted of regulatory T cells to Rag2-deficient adult mice induced marked inflammation at the colon mucosa and strong accumulation of CD44^hi^ CD4^+^ T lymphocytes (Fig. 4c,d). In contrast, adult LP CD4^+^ T lymphocytes failed to evoke inflammation consistent with their mature phenotype. Thus, the sustained immature phenotype of neonatal intestinal CD4^+^ T cells does not appear to result from T-lymphocyte-intrinsic mechanisms but to depend on the local neonatal tissue environment.

Next, we aimed at the identification of conditions that alter the local neonatal tissue milieu and facilitate early CD4^+^ T-cell maturation. First, we employed invasive and non-invasive enteric infection models. Oral infection with the enteropathogenic microorganisms rotavirus and *Salmonella enterica* destroy the epithelial barrier integrity and induce potent innate immune stimulation^32^,^34^,^35^. In contrast, infection of newborn mice with the non-invasive pathogen *Giardia lamblia* does not cause overt epithelial barrier disruption and mucosal tissue damage despite abundant colonization of the small intestine (own unpublished observation and refs 36, 37). Clearly, the fraction and absolute number of CD44^hi^ neonatal CD4^+^ T cells in the small intestine increased significantly on *Salmonella* or rotavirus infection, whereas infection with *G. lamblia* had no significant effect (Fig. 4e and Supplementary Fig. 3b). Consistent with previous results^38^, TCRαβ^+^ T cells also conferred significant protection after rotavirus infection of neonates illustrating their potency to exert effector function (Fig. 4f). Second, we tested the emergence of CD44^hi^ antigen-specific CD4^+^ cells in mice after high-dose oral antigen challenge in TCR transgenic neonates. Ovalbumin was administered daily at high doses (10 mg per day over 6 days starting at day 3 after birth) by oral gavage to DO11.10 neonates bearing an OVA-specific TCR. The proportion of CD44^hi^CD62L^−^ T cells in Peyer's patches and the percentage of total intestinal cells expressing high levels of CD44 were significantly increased after ovalbumin administration compared with littermate controls given PBS (Fig. 4g and Supplementary Fig. 3c,d). This finding was also observed in OTII mice expressing a transgenic TCR with different ovalbumin specificity (Supplementary Fig. 3e). Restimulated CD4^+^ OTII lymphocytes isolated from antigen-exposed neonates, however, failed to produce enhanced amounts of IL-17 and IFN-γ (Supplementary Fig. 3f). To confirm that OVA-mediated activation occurred in an antigen-specific manner, OTII T lymphocytes were transferred into wt neonates facilitating the comparative analysis of CD44 expression on OTII and endogenous T lymphocytes (Supplementary Fig. 3g). Thus, both, invasive infection and exposure to high antigen concentrations are able to induce rapid maturation of neonatal CD4^+^ T cells. These results illustrate that although neonatal CD4^+^ T lymphocytes remain naïve for a sustained period of time under conditions of postnatal host–microbial homeostasis, they are intrinsically functional and rapidly mature under conditions of mucosal stress or excessive antigen exposure. The functional outcome of neonatal CD4^+^ T-lymphocyte stimulation under these conditions, however, requires further investigation.

### Active maintenance of CD4^+^ T cell immaturity

**Figure 5:**
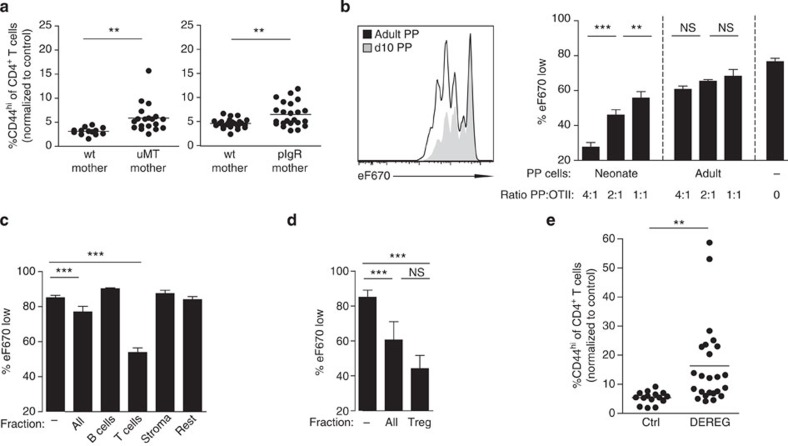
Mechanisms that maintain immaturity of intestinal CD4 T cells during the homeostatic postnatal period. (**a**) Percentage of CD44^hi^ CD4 T cells in the SI of 11-day-old B-cell-sufficient (μMT^+/−^ or μMT^+/+^) (left panel) and IgA-sufficient (pIgR^+/−^ or pIgR^+/+^)(right panel) neonates fed by B-cell or IgA-sufficient (wt mother) or deficient (μMT^−/−^ or pIgR^−/−^ mother) dams. (μMt: *n*=4 litters from four experiments; pIgR: *n*=2 litters from two experiments, mean; unpaired Student's *t*-test, **P<0.01, ***P<0.001). (**b**) Comparative proliferation assay culturing OVA-loaded BMDCs, eFluor670-labelled OTII cells together with neonatal or adult PP cells for 3 days at the indicated ratio (ratio PP:OTII; *n*=4 technical replicates, representative of five similar independent experiments, mean±s.d.; one-way ANOVA, Bonferroni's post test, ***P<0.001). (**c**) Comparative proliferation assay culturing OVA-loaded BMDCs, and eFluor670-labelled OTII T lymphocytes together with FACS-sorted subgroups of neonatal PP cells for 3 days at the ratio of 4:1 (PP:OTII: B and T cells) or 2:1 (PP:OTII: stroma and rest). (*n*=3–4 technical replicates, representative of four similar independent experiments, mean±s.d.; one-way ANOVA, Bonferroni 's post test, ***P<0.001). (**d**) Comparative proliferation assay culturing OVA-loaded BMDCs, eFluor670-labelled OTII T lymphocytes together with FACS sorted neonatal regulatory T cells (T_Reg_, from Foxp3 reporter mice) for 3 days at the ratio of 4:1 (ratio PP:OTII). (*n*=2 replicates from two experiments with T_Regs_ pooled from 20 and 8 neonates per experiment, respectively, mean±s.d.; one-way ANOVA, Bonferroni 's post test, ***P<0.001, NS, not significant). (**e**) Percentage of CD44^hi^ cells among CD4 T lymphocytes (using non-transgenic littermate controls as a reference gate) in the SI of 11-day-old DEREG mice and non-transgenic littermate controls all treated with DT on days 1/2/5/6 (*n*=5 litters from five experiments, mean; unpaired Student's *t*-test, **P<0.01.

We next aimed at identifying mechanisms that actively suppress CD4^+^ T-cell maturation in the neonate intestine under homeostatic conditions. First, we examined antigen uptake by neonatal antigen-presenting cells, since antigen presentation is a prerequisite for T-cell activation. MHCII^+^CD11b^+^ antigen-presenting cells were present in the neonate intestine (Fig. 1b). Also, orally administered fluorophore-conjugated antigen was rapidly (<4 h) delivered from the intestinal lumen and internalized by MHCII^+^ myeloid cells of the neonatal small intestinal mucosa (Supplementary Fig. 4a). Since antigen uptake and presentation by antigen-presenting cells seemed unaffected in neonates, we next sought to test whether antigen sequestration by maternal immunoglobulins that are abundantly present in breast milk might contribute to sustained naïveté of neonatal small intestinal CD4^+^ T cells. We therefore crossed B-cell-deficient homozygous μMT^−/−^ dams and μMT^+/+^ sires. The B-cell-proficient heterozygous (μMT^+/−^) offspring exhibit a normal B-cell compartment but lack exposure to maternal breast milk-transferred immunoglobulins. Indeed, heterozygous μMT neonates from homozygous B-cell-deficient μMT dams harboured enhanced numbers of CD44^hi^CD62L^low^ expressing intestinal CD4^+^ T cells in Fig. 5a and Supplementary Fig. 4b,c. We additionally analysed heterozygous neonates from pIgR-deficient dams that produce immunoglobulins but are unable to secrete polymeric immunoglobulins into the breast milk^39^. Again, neonates from these animals exhibited an enhanced fraction of CD44^hi^ expressing intestinal CD4^+^ T cells (Fig. 5a and Supplementary Fig. 4c). This suggests that antibodies in breast milk, in particular SIgA, restrict the level of exposure to antigens and contribute to the maintenance of immature intestinal CD4^+^ T cells in the neonate host.

In addition, we examined cell-mediated mechanisms of T-cell suppression. Systemic depletion of CD71^+^ erythroid cells, which has previously been implicated in the negative regulation of neonatal immune stimulation^24^, did not result in enhanced numbers of CD44^hi^ neonatal intestinal CD4^+^ T cells (Supplementary Fig. 4d). In contrast, tissue-resident cells derived from dissected PPs from 11-day-old neonates but not adult mice were able to significantly and dose dependently inhibit the proliferation of CD4^+^ OTII lymphocytes following co-incubation with OVA-loaded DCs *in vitro* (Fig. 5b). Separation of neonatal Peyer's patch cells into TCRβ^+^, CD19^+^, CD45^−^ and TCRβ^−^ CD19^−^CD45^+^ cells by fluorescence-activated cell sorting (FACS) sorting and co-incubation with CD4^+^ OTII lymphocytes and OVA-loaded DCs identified the strongest inhibitory effect for the TCRβ^+^ T-cell subset (Fig. 5c). Subsequent isolation of the regulatory T-lymphocyte (T_Reg_) population from the TCRβ^+^ fraction revealed their dominant function for T-lymphocyte suppression (Fig. 5d). To demonstrate the critical role of T_Regs_ *in vivo*, we employed the DEREG mouse model that allows the inducible depletion of T_Regs_ by administration of diphtheria toxin (DT). Indeed, intestinal CD4^+^ T lymphocytes isolated from DT-treated DEREG neonates exhibited significantly enhanced absolute and relative numbers of CD44^hi^ cells (Fig. 5e and Supplementary Fig. 4e). These results identify the critical role of neonatal T_Regs_ in the suppression of intestinal CD4^+^ lymphocyte maturation. The suppressive function of neonatal T_Reg_ cells was independent of the production of IL-10 (Supplementary Fig. 4f). This observation is consistent with cell–cell proximity-dependent inhibition of cell proliferation (Supplementary Fig. 4g). Thus, IgA-mediated restriction of antigen translocation and T_Regs_ in the neonate Peyer's patch contribute significantly to active suppression of neonatal CD4^+^ T-lymphocyte maturation.

## Discussion

Recent reports have impressively documented the critical role of the enteric microbiota in driving the maturation of the adult mucosal immune system. In the absence of microbiota, adult mice exhibit reduced size of PPs lacking germinal centres, smaller isolated lymphoid follicles and fewer CD4^+^ and CD8^+^ T lymphocytes as well as IgA^+^ plasma cells^40^. Vice versa, adult conventional mice exhibit lower numbers of immature CD4^+^ T lymphocytes in the small intestine than germ-free mice. Individual commensal bacteria significantly contribute to this scenario. For example, segmented filamentous bacteria (SFB) promote the maturation of the gut mucosal barrier and induce bacteria-specific TH17 cells^41^,^42^. This situation seems to be substantially different in the neonate intestine. Our results demonstrate that intestinal CD4^+^TCRαβ^+^ T cells in the homeostatic neonate intestine exhibit a sustained immature phenotype despite the rapid emergence of a dense and highly dynamic enteric microbiota. We show that neonatal CD4^+^ T cells are not intrinsically non-responsive to immune stimulation. Instead, local age-dependent mechanisms actively suppress immune stimulation and maintain a low maturation status of CD4^+^ T cells throughout the postnatal period.

Per definition, T lymphocytes in the neonatal intestine can be considered recent thymic emigrants (RTE), progenitors of mature naïve T lymphocytes^43^,^44^. In comparison with mature naïve T lymphocytes, RTEs were described to exhibit functional deficits such as reduced survival^45^,^46^, cytokine secretion and cytolytic activity^47^. Impaired T-cell priming was explained by reduced CD28 expression and co-stimulation despite higher TCR and CD3 expression in these cells^27^. Whereas co-incubation with mature naïve T cells has been reported to restore function of CD4^+^ RTEs, the functional abilities of CD4^+^ RTEs in the absence of mature T lymphocytes in the neonate host have not been systematically investigated^46^. CD4^+^ RTEs contribute to the TH2 bias in the neonate host by early secretion of IL-4 and IL-13 (ref. 48). IL-4 and IL-13, in the absence of IL-12, cause TH1 cell depletion on secondary antigen exposure^49^. Our results demonstrate that intestinal CD4^+^ RTEs in fact possess the intrinsic ability to rapidly gain maturity and effector function *in vitro* or when transferred to an adult host, exposed to high antigen concentrations or challenged with an invasive infectious agent^50^. Both in mice and humans, RTEs preferentially home to preformed lymphoid structures^51^,^52^. Entry into lymphoid organs such as spleen and lymph nodes and contact with dendritic cells was shown to be required for subsequent cell maturation^53^. We show that T-lymphocyte homing in the newborn is independent of microbial exposure and innate or adaptive immune stimulation, but relies on β7 integrin expression. α4β7 integrin on naïve lymphocytes interacts with the mucosal addressin cell adhesion molecule (MAdCAM)-1 on mucosal endothelial cells of PPs, MLNs and the LP^54^,^55^,^56^. α4β7 expression is further enhanced by contact with CD103^+^ dendritic cells exposed to retinoic acid found in food or breast milk^57^. Consistently, β7 integrin-deficient mice, similar to MAdCAM-1-deficient mice, show severely impaired lymphocyte recruitment and PP development^58^.

Our results identify mechanisms that facilitate sustained immaturity of intestinal CD4^+^ T cells during the postnatal period. Maternal SIgA and neonatal T_Reg_ cells act in concert to prevent postnatal CD4^+^ maturation under homeostatic conditions. Breast milk-derived SIgA might reduce translocation of luminal antigens previously encountered by the dam and thus prevent immune stimulation by environmental antigens^39^,^59^. In accordance, exposure of neonates to novel antigens not previously seen by the maternal immune system (for example, ovalbumin or infectious agents) resulted in T-cell effector memory generation. In addition, we found that T_Reg_ cells within the neonate's Peyer's patches inhibit intestinal CD4^+^ T-lymphocyte maturation. This effect requires cell–cell proximity and acts locally in the neonatal patch environment in accordance with the rapid acquisition of effector function of neonatal lymphocytes after transfer into adult Rag2-deficient animals. The characterization of the precise underlying molecular mechanism(s) requires further investigation. Our *in vivo* results exclude a significant contribution of IL-10 in accordance with the required cell proximity and the debated role of IL-10 for thymus-derived T_Regs_ that dominate in the neonate^60^. Additional mechanisms proposed to be involved in T_Reg_-mediated lymphocyte control are the secretion of other inhibitory cytokines such as TGFβ or IL-35, direct cytolytic activity via granzyme and perforin, metabolic starvation of the essential signal IL-2 or adenosine as well as indirect inhibitory circuits on DC function via CTLA4 or LAG3 (ref. 61). Despite these unresolved questions, the critical importance of neonatal T_Regs_ to maintain tolerance to self-antigen during adult life has been demonstrated^16^,^62^.

Delayed maturation of T lymphocytes during early postnatal development has also been described in humans^63^,^64^. Reduced acquisition of T-lymphocyte effector function during the first months of life was associated with a decreased capacity in antigen presentation^63^ and the presence of a distinct, more tolerogenic fetal T-cell lineage^64^. The situation in the neonate intestine therefore resembles the setting in systemic organs more strongly than in the adult intestine, where immune effector and regulatory functions in a ‘controlled inflammatory reaction' maintain host–microbial homeostasis^7^. Also, the situation in the neonate murine intestine is strikingly different from the situation in the neonate lung, where early microbial exposure drives induction of regulatory T cells to prevent exaggerated airway responsiveness during adulthood^15^.

What might be the host's benefit from sustained immaturity of neonatal intestinal CD4^+^ T cells? First, neonatal T lymphocytes have been shown to exhibit lower average avidity but enhanced cross-reactivity^43^,^65^. The enhanced cross-reactivity is linked to a lack of terminal deoxynucleotidyl transferase expression early after birth and absence of *N* nucleotide addition during V-(D)-J gene recombination^1^. Active suppression of neonatal T lymphocytes might thus protect from microbiota-stimulated maturation of cross-reactive T lymphocytes and autoimmunity. Likewise, homeostatic proliferation of T cells was demonstrated to promote the development of autoimmunity^66^. Second, post-thymic deletion of autoreactive T cells was demonstrated in secondary lymphoid organs^67^. Active suppression of T-cell maturation might thus provide a time window in the neonate intestine to reinforce self-tolerance. Third, mucosal antigens in the neonatal intestine are only transiently present. Both nutrient antigens derived from breast milk as well as microbial constituents of the early commensal bacteria are replaced after the postnatal period by solid food antigens and the markedly altered, complex bacterial composition of the post-weaning microbiota. Thus, restriction of T-lymphocyte maturation during the postnatal period might prevent the expansion of lymphocyte clones with ‘neonatal' reactivity that fail to support host–microbial homeostasis and provide protection during adulthood. This might preserve the broad TCR repertoire to adulthood when antigens become available that ultimately play a significant role throughout adult life. Further studies are needed to examine the functional consequences of early T-lymphocyte immaturity for mucosal immune homeostasis. The models used here, however, induce maturation of a relatively small fraction of T cells only. Conditions that lead to broad maturation of T cells need to be identified to characterize the downstream consequences of early neonatal T-lymphocyte maturation.

In conclusion, we provide the first systematic analysis of the development of the major immune cell subsets during the postnatal period in the murine small intestine. We observe postnatal immune stimulation-independent intestinal recruitment of CD4^+^ T lymphocytes. Neonatal CD4^+^ T cells rapidly acquire a distinct gene expression profile but remain immature throughout the postnatal period despite antigen exposure and the rapid emergence of the microbiota. We identify the mutual contribution of maternal IgA and neonatal T_Regs_ in the suppression of neonatal T lymphocytes. Our results thereby support the concept of the ‘neonatal window' that represents a particularly sensitive period in the ontogeny of the host's immune system. Furthermore, our data illustrate the marked differences between the neonate and adult mucosal immune system and indicate that strategies beneficial to the adult host might not necessarily also help the neonatal organism.

## Methods

### Ethic statement

All animal experiments were performed in compliance with the German animal protection law (TierSchG) and approved by the local animal welfare committee Niedersaechsisches Landesamt fuer Verbraucherschutz und Lebensmittelsicherheit Oldenburg, Germany). Mice were housed under specific pathogen-free conditions and handled in accordance with regulations defined by FELASA and the national animal welfare body GV-SOLAS (www.gv- solas.de/index.html).

### Animals

Conventionally raised C57BL/6, Tlr4^−/−^, Nod2^−/−^, MyD88^−/−^, Trif^Lps2/Lps2^, Ccr9^−/−^, Integrin β 7^−/−^, TCRα^−/−^, Rag2^−/−^, FoxP3eGFP, μMt^−/−^, pIgR^−/−^, OTII, DO11.10 transgenic mice as well as germ-free mice were bred at the Hannover Medical School Animal Facility and were checked daily for litters. Indu-*Rag1* mice^33^ and Rag-GFP (Foxp3-hCD2 × RAG1-GFP, generously provided by Shohei Hori, RIKEN, Saitama, Japan) mice were kept at the animal facility of the Helmholtz Centre for Infection Research. DEREG mice were bred at the TWINCORE animal facility. Male and female mice were used in all age groups. Gastric gavage of neonate mice was carried out using a 24G silicon catheter (Vygon, Aachen, Germany).

### Cell isolation

For the isoloation of small intestinal lymphocytes, small intestines were removed, opened longitudinally and cleaned from luminal contents. Tissue was digested in Liberase (Roche)/DNAse I (Roche)/10% FCS/RPMI at 37 °C for 45 min for neonates (< day 15) and 2 × 45 min for older mice and subsequently separated on a 40%/70% Percoll gradient. For colonic LP cell isolation, the epithelial compartment was removed by 3 × 15 min incubation in EDTA/HBSS before Liberase TM digest. For Peyer's patch cell isolation, Peyer's patches were excised using the binocular for neonates and pressed trough a mesh for subsequent FACS analysis including CD62L staining or digested in Liberase TM (Roche)/DNAse I (Roche)/10% FCS/RPMI at 37 °C for 1.5 h for further *in vitro* culture or restimulation for intracellular cytokine staining. To obtain a single-cell suspension of leukocytes, spleens were pressed through a mesh and treated with erythrocyte lysis buffer for 10 min at RT.

### Flow cytometry and cell sorting

The following fluorophore- or biotin-conjugated antibodies specific for mouse surface antigens (eBioscience, BioLegend or BD) were used for FACS analysis and cell sorting: TCRβ-PE/PE Cy7 (30-F11, 1:100), CD4-PerCP Cy5.5 (RM4–5, 1:200), CD8α-APC Cy7 (53–6.7, 1:400), TCRδ-PE Cy7 (GL3, 1:100), CD19-APC (6D5, 1:100), CD11b-FITC (M1/70, 1:200), CD11c-APC Cy7 (N418,1:100), MHCII-PE (AF6–120.1, 1:400), F4/80-APC (BM8, 1:100), Siglec F-PE (E50–2440, 1:100), CD44-APC (IM7, 1:100), CD62L-PE (MEL-14, 1:100), CD69-PerCP (H1.2F3, 1:100) Vα2-PE (B20.1, 1:200), CD45 (104, 1:200), CD8β-Cy5 (homemade, 1:100), IFNγ-PE Cy7 (XMG1.2, 1:100) and IL-17A-APC (eBio17B7, 1:100). FACS acquisition was performed on LSRII (BD) and analysed with FlowJo (Treestar). All samples were pregated on CD45+DAPI^−^ (4′,6-diamidino-2-phenylindole) singlets. Cell sorting was performed on FACS Aria (BD) (microarray) or XDP MoFlow (Beckmann Coulter; T-cell transfer colitis and OTII *in vitro* proliferation assays).

### Cell stimulation and cytokine measurement

For intracellular cytokine detection, cells were stimulated with phorbol 12-myristate13-acetate (PMA)/Ionomycin (50 ng ml^−1^ and 2 μg ml^−1^, respectively) in the presence of Brefeldin A for 4 h. Intracellular staining was performed using the BD Cytofix/Cytoperm kit. For detection of cytokines in the supernatant, CD4+TCRβ+ lymphocytes from neonatal and adult PPs were FACS sorted to 10^5^ cells per well and restimulated with PMA/Ionomycin in 100 μl medium overnight. Supernatants were collected and cytokines were detected using the mouse Th1/Th2/Th17/Th22 13plex FlowCytomix kit from eBioscience.

### Immunostaining and histology

Staining with TCRβ-bio (eBioscience, 30-F11) and EpCAM-APC (eBioscience, 48.8) was performed on 8-μm sections obtained from freshly frozen OCT compound (Tissue-Tek) embedded tissue. Sections were dried and fixed using methanol at −20 °C for 10 min followed by rehydration in PBS for 15 min. Consecutive blocking with the Avidin/Biotin blocking kit (Vector) and 5% goat serum in TBST was performed before immunostaining. For TCRβ detection, the TSA Cy3 system (Perkin Elmer) was used. Staining with MadCAM (BioLegend, MECA-367) and CD31FITC (BD, MEC13.3) was performed on 8-μm sections obtained from freshly frozen OCT compound (Tissue-Tek)-embedded tissue. Sections were fixed in acetone at −20 °C for 10 min followed by rehydration in TBST for 15 min. Slides were mounted in Vectashield (Vector) supplemented with DAPI and pictures were taken with an Apo-Tome equipped Axioplan-2 microscope connected to a digital camera (Zeiss). Haematoxylin and eosin staining of paraformaldehyde-fixed paraffin-embedded tissue sections was performed according to Mayer's protocol using reagents from Roth.

### Microarray analysis

Microarray analysis was performed in quadruplicates. For neonatal samples (6 and 11 days after birth), tissues from all neonates of one litter were pooled to yield enough material. CD4^+^TCRβ^+^CD8α^−^CD45^+^DAPI^−^ cells (0.5–2 × 10^5^) from indicated organs were sort-purified and RNA was isolated using an RNeasy Micro Kit (Qiagen). The microarray study was performed by use of a refined version of the Whole Mouse Genome Oligo Microarray 4 × 44 K v2 (Design ID 026655, Agilent Technologies), called ‘026655AsQuadruplicatesOn180k' (Design ID 048306) developed in the Research Core Unit Transcriptomics of Hannover Medical School. Microarray design was defined at Agilent's eArray portal using a 4 × 180 k design format for mRNA expression as template. All non-control probes of design ID 026655 have been selected to be printed four times onto one 180 k Microarray (on-chip quadruplicates). Control probes required for proper Feature Extraction software algorithms were determined and placed automatically by eArray using recommended default settings. Total RNA (4–8 ng) was used to prepare aminoallyl-UTP-modified (aaUTP) cRNA (Amino Allyl MessageAmp II Kit; #AM1753; Life Technologies) as directed by the company (applying one round of amplification). The labelling of aaUTP-cRNA was performed by use of Alexa Fluor 555 Reactive Dye (#A32756; LifeTechnologies). Before the reverse transcription reaction, 1 μl of a 1:50,000 dilution of Agilent's ‘One-Colour spike-in Kit stock solution' (#5188–5282, Agilent Technologies) was added to each total RNA sample. cRNA fragmentation, hybridization and washing steps were carried out as recommended in the ‘One-Colour Microarray-Based Gene Expression Analysis Protocol V5.7', except that 45 ng of each fluorescently labelled cRNA population were used for hybridization. Slides were scanned on the Agilent Micro Array Scanner G2565CA (pixel resolution 3 μm, bit depth 20). Data extraction was performed with the ‘Feature Extraction Software V10.7.3.1' using the extraction protocol file ‘ GE1_107_Sep09.xml'. Extracted raw data were imported into Omics Explorer software v3.0 (Qlucore) under default import settings for Agilent One-Colour mRNA Microarrays. Accordingly, data processing steps were: (1) removal of control measurements, (2) log base 2 transformation, (3) normalization of non-control values by shifting to 75 Percentile, (4) averaging of values from on-chip replicates and (5) baseline transformation to the median.

### T-cell transfer colitis

A modified version of the T-cell transfer colitis model was used^68^. In brief, GFP^+^ T regulatory (T_Reg_)-depleted small intestinal CD4^+^ T cells from 11 days old neonate and adult FoxP3eGFP animals were sorted and 1–4 × 10^4^ cells per recipient were transferred intraperitoneally (i.p.) into 6–8-week-old Rag2-deficient males. Weight was monitored and the complete group of mice was killed when the first mouse had lost 20% of the initial weight. Disease was evaluated by histology and FACS analysis of CD4 T-cell infiltration into the colonic LP.

### Infection models

Rotavirus infection was performed as previously published^69^. For that, 4-day-old neonates were infected orally with 10^5^ × ID_50_ of the murine rotavirus strain EDIM (provided by Lennart Svensson, Linköping, Sweden) in 5 μl PBS. Antigen enzyme-linked immunosorbent assay (ELISA) from colon homogenates 8 days post infection (d.p.i.) was performed to ensure successful infection using the RIDASCREEN Rotavirus Elisa Kit (R-Biopharm).

For infection with *Salmonella enterica subsp. enterica serovar* Typhimurium ATCC14028 (NCTC12023) wild-type (WT)^34^ bacteria were cultured in Luria Bertani (LB) broth overnight at 37 °C, diluted 1:10 and incubated at 37 °C until reaching the logarithmic phase (OD_600_ ∼0.5). Bacteria were washed and adjusted to OD_600_ 0.55–0.60 containing ∼1.5–2.0 × 10^8^ c.f.u. ml^−1^ and diluted to obtain the appropriate infection dose. Four-day-old neonates were infected orally with 2 × 10^3^ c.f.u. of *S. typhimurium* in a volume of 2 μl PBS. The infection status was assessed by serial dilution and plating of *Salmonella* and gross pathology examination of the peritoneal organs.

*G. lamblia* trophozoites were cultured axenically in *G. lamblia* TYI-S-33 Medium. Four-day-old neonates were infected with 2 × 10^5^ *Giardia lamblia* trophozoites (GB strain, ATCC 50581) in 20 μl PBS by gastric gavage. Mice were killed 8 d.p.i. and small intestines were collected for analysis. *G. lamblia* infection was verified in small intestinal homogenates by trophozoite counting and antigen detection via ELISA (IBL International).

### OTII cell *in vitro* proliferation assay

eFluor670-labelled OTII cells were co-cultured with OVA-loaded, activated BMDCs (200 μg ml^−1^ Ova (Grade VI, Sigma) and 0.5 μg ml^−1^ lipopolysaccharide)^70^ at a ratio of 50:1 and a ratio of 4:1 of PP cells to OTII cells—unless otherwise indicated—for 3 days at 37 °C with 5% CO2. For the transwell experiments, the cells were co-cultured with or without a transwell (DCs and OTII cells in the lower compartment, PP cells in the upper compartment; 4 μm, Millicell). The ratio of PP cells to OTII cells to DCs was 20:4:1.

### PP depletion

PPs were depleted by injecting 2 mg anti-IL7Rα or isotype control antibody (A7R34 and 2A3, BioXCell) into timed-pregnant dams at gestation E14.05 (ref. 20).

### Depletion of TRegs

T_Regs_ were depleted as previously described^16^. In brief, newborn DEREG mice and non-transgenic littermate controls were injected i.p. with 25 and 100 ng DT (Merck) on days 1/2 and 5/6 after birth, respectively.

### OTII cell transfer

Neonatal B6N mice were injected i.p. with 1–2*10^5^ MACS purified congenic OTII cells (isolated from adult mice) on days 2 and 3 after birth. Subsequently, they were gavaged 10 mg OVA or PBS daily at days 4–9 after birth. PP lymphocytes were analysed at day 11 after birth.

### CD71^+^ cell depletion

Neonatal mice were injected with 280 and 420 μg anti-CD71 or isotype control (R17 217.1.3/TIB-219 and 2A3, BioXCell) on days 6 and 8 after birth, respectively. Mice were killed at 9 days of age. Depletion efficiency was correlated to signs of anaemia in the spleen.

### Statistical analysis

Group sizes were estimated according to a presumed s.d. and an expected type one error of <0.05. Depending on the initial results, the sample size in subsequent experiments was adjusted accordingly. Within all figures, each data point reflects results from a single mouse unless otherwise indicated; in *in vitro* co-culture experiments each data point represents a technical replicate. Statistical analysis was performed using GraphPad Prism software. For comparison of unmatched two groups, the unpaired Student's *t*-test was used. For comparison between three or more groups one-way ANOVA followed by Bonferroni's post test was used unless otherwise specified in the figure legend. All data are presented as mean or mean±s.d. and *P*<0.05 are considered as significant. In all figures, *P<0.05; **P<0.01; ***P<0.001; NS, not significant.

## Additional information

**Accession codes**: Microarray data have been deposited at GEO database under accession code GSE60515.

**How to cite this article:** Torow, N. *et al.* Active suppression of intestinal CD4^+^TCRαβ^+^ T-lymphocyte maturation during the postnatal period. *Nat. Commun.* 6:7725 doi: 10.1038/ncomms8725 (2015).

## Supplementary Material

Supplementary Information Supplementary Figures 1-4
