# Supplementary material for: Active suppression of intestinal CD4+TCRαβ+ T-lymphocyte maturation during the postnatal period
Source: Nat Commun. 2015 Jul 21;6:7725. doi: 10.1038/ncomms8725 (PMC4518322; doi:10.1038/ncomms8725)
Supplement: Supplementary Information — Supplementary Figures 1-4 [file ncomms8725-s1.pdf]

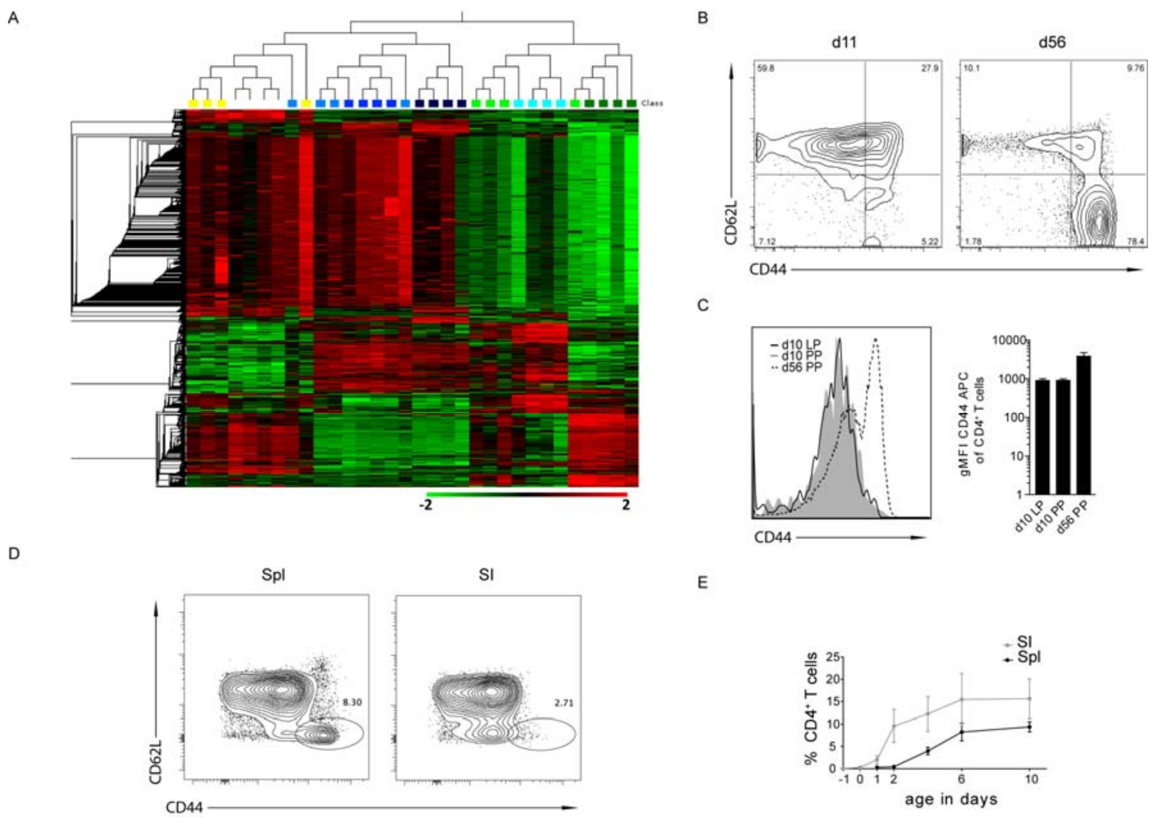

Supplementary Figure 1: Maturation status of neonatal mucosal CD4 T lymphocytes. (a) Transcriptome analysis of flow cytometry-sorted $CD4^{+}CD8^{-}$ $TCR\beta^{+}$ cells isolated from the indicated organs at the indicated ages (days, d). The array was performed in quadruplicates (n=4) with each group comprising sorted cell populations pooled from one litter for neonate animals (d6 and day 11) and 2-3 adult mice per sample. Thymus (Th) d6, small intestine (SI) d6, SI d11, mesenteric lymph node (mLN) d6, mLN d56, lamina propria (LP) d28, LP d56 and Peyer's patch (PP) d56. Hierarchical clustering of the samples (upper row) and hierarchical clustering of 10,000 differentially regulated individual genes using multigroup comparison (ANOVA analysis) and a false discovery rate (FDR) of $<10^{4}$. **(b)** Representative FACS plot of surface CD62L and CD44 expression on $CD8\alpha\beta^{+}$ T cells from Peyer's patches from 11- and 56-days old mice **(c)** Flow cytometric analysis (left panel) and quantification (right panel) of CD44 expression levels on an enriched *lamina propria* fraction of CD4 T lymphocytes isolated from 11-day-old (d10 LP) neonates in comparison to CD4 T lymphocytes from neonatal (d10 PP) and adult (d56 PP) Peyer's patches. (n=4, mean±SD). **(d)** Representative FACS plot of surface CD62L and CD44 expression on CD4 T cells from Peyer's patches and spleen from 11-day-old mice. **(e)** Kinetic of CD4 T cell recruitment to the spleen and small intestine (SI) during the postnatal period. CD4 T cells are shown as percent of total $CD45^{+}$ immune cells (n=4-11, mean±SD).

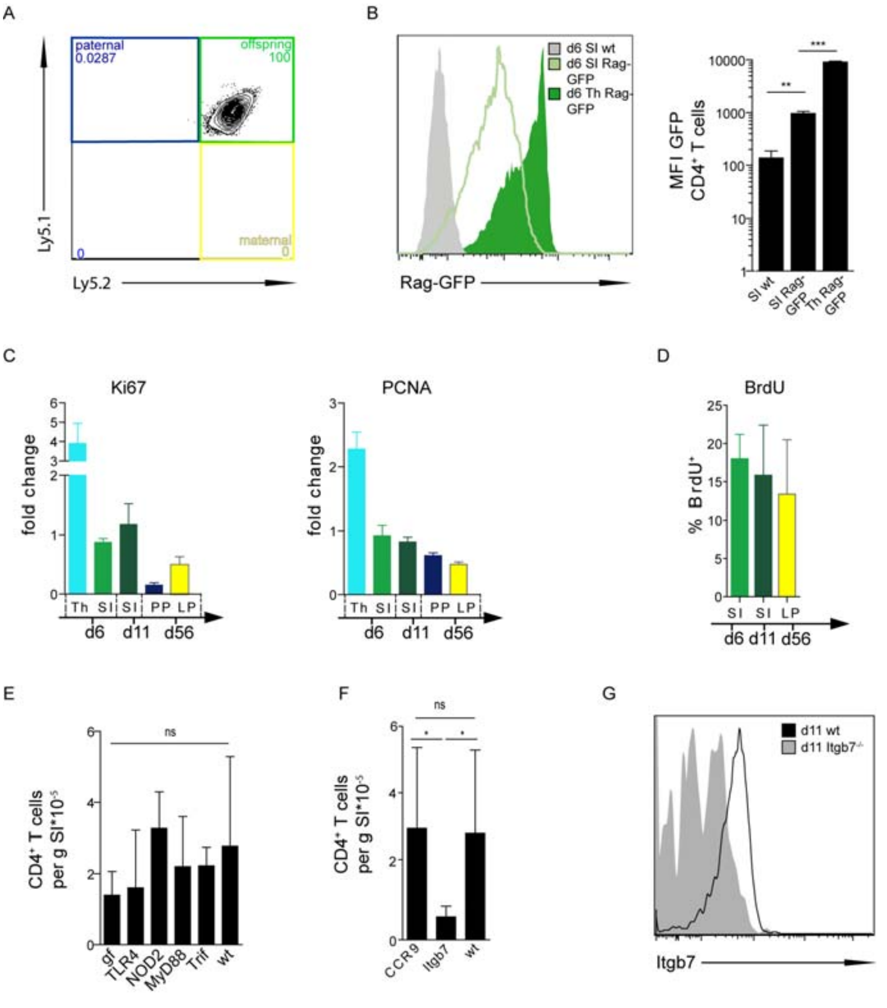

Supplementary Figure 2: Origin of neonatal mucosal CD4 T lymphocytes. (a) Flow cytometric analysis of the congenic markers Ly5.1 and Ly5.2 on CD4 T lymphocytes isolated from the small intestine of 6-day-old heterozygous neonates (paternal marker: Ly5.1, maternal marker: Ly5.2). (n=2 litters in 2 independent experiments) **(b)** Flow cytometric analysis (left panel) and quantification (right panel) of the GFP expression levels in CD4^{+} T lymphocytes isolated from the small intestine (SI) and thymus (Th) of 6-day-old Rag-Gfp neonates or the small intestine of age-matched wildtype (wt) control animals. (n=4-6, representative of 2 independent experiments, mean±SD; one-way ANOVA, Bonferroni's post test, **, p<0.01, ***, p<0.001). **(c)** Fold change values of mRNA expression (normalized to the overall mean intensity of all samples) of the proliferation markers Ki67 and PCNA measured by microarray analysis in sorted CD4 T lymphocytes from thymus (Th, light blue), small intestine (SI) of 6- day-old (light green) and 11-day-old (dark green) mice as well as Peyer's patches (PP, dark blue) and *lamina propria* (LP, yellow) of 56-day-old mice. (n=4, mean±SD). **(d)** Percentage of BrdU^{+} cells of CD4 T lymphocytes recovered from the small intestine (SI) of 6-day-old (light green) or 11-day-old (dark green) or lamina propria (LP, yellow) of 56-day-old mice 20h after an i.p. injection of 80 mg/kg BrdU. (n=4, mean±SD). **(e)** Comparative analysis of the absolute numbers of CD4^{+}TCRβ^{+} T lymphocytes normalized to tissue weight in intestinal tissue of germ free (gf), Tlr4$^{-/-}$, Nod2$^{-/}$, MyD88$^{-/-}$, Trif$^{Lps2/Lps2}$ and wildtype (wt) 11-day-old mice (n=4-13 pooled from 2 experiments, mean±SD; one-way ANO VA, Bonferroni 's post test, ns, not significant). **(f)** Comparative analysis of the absolute numbers of CD4^{+}TCRβ $^{+}$ T lymphocytes normalized to tissue weight in intestinal tissue of Ccr9$^{-/-}$, Itgb7$^{-/-}$ and wildtype (wt) 11-days-old mice (n=8-13, mean± SD; one-way ANOVA, Bonferroni 's post test, **, p<0.01, ns, not significant). **(g)** Flow cytometric analysis of the expression level of integrin β7 on CD4^{+} T lymphocytes isolated from small intestinal (SI) tissue of 11-day-old wildtype (wt) and Itgb7$^{-/-}$ neonate mice (n=6).

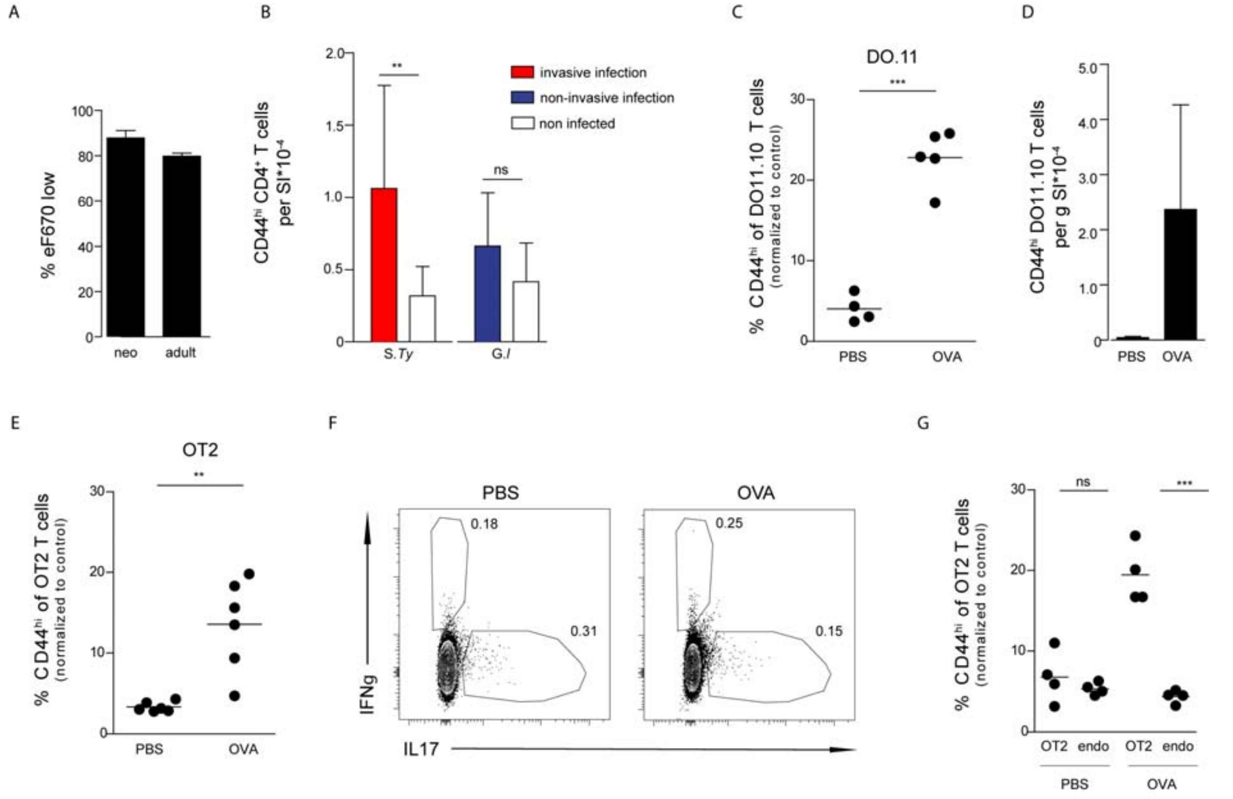

Supplementary Figure 3: Maturation of neonatal CD4 T lymphocytes following mucosal challenge. **(a)** Proliferation status of *ex vivo* activated $CD4^+$ T cells isolated from Peyer's patches from SI of 11- and 56-days-old mice after 3 days of culture in presence of antiCD3/CD28 beads. **(b)** Comparative analysis of the absolute number of $CD44^{hi}CD4^+TCR\beta^+$ T lymphocytes in intestinal tissue of neonate mice infected with *S.* Typhimurium (infected at day 4 post parturition (pp) and analyzed at 4 dpi) and *Giardia lamblia* (infected at day d4 pp and analyzed at 8 dpi) (Cell numbers were not normalized to tissue weight due to pathologically increased weight in *S.Typhimurium* infected neonates (n=4; representative of two independent experiments, mean±S D; unpaired Student's t test (each infection model represents an independent experiment type, therefore not ANOVA) *, p<0.05, ***, p<0.001; ns, not significant). Percentage **(c)** and absolute numbers **(d)** of $CD44^{hi}$ CD4 T lymphocytes in the small intestine of 11-day-old DO11.10 neonates after daily gavage of 10 mg ovalbumin (OVA) starting at day 3 pp (right panel) (n=8 from two experiments, mean± SD; unpaired Student's t test, **, p<0.001). **(e)** Percentage of $CD44^{hi}$ CD4 T lymphocytes in the small intestine of 11-day-old OTII neonates after daily gavage of 10 mg ovalbumin (OVA) starting at day 3 after birth (right panel). (n=6 from two experiments, mean; unpaired Student's t test, **, p<0.01). **(f)** Intracellular FACS staining for IFNγ and IL17-A of OTII cells isolated from Peyer's patches of 11-day-old OTII neonates restimulated with PMA/Ionomycin after daily gavage of 10 mg ovalbumin (OVA) or PBS starting at day 3 after birth (n=4). **(g)** Percentage of $CD44^{hi}$ cells among OTII and endogeous CD4 T lymphocytes in the small intestine of 11-day-old wt neonates after transfer of OTII cells and daily gavage of 10 mg ovalbumin (OVA) or PBS starting at day 3 after birth (n=4, mean; one-way ANO VA, Bonferroni 's post test, ***, p<0.001).

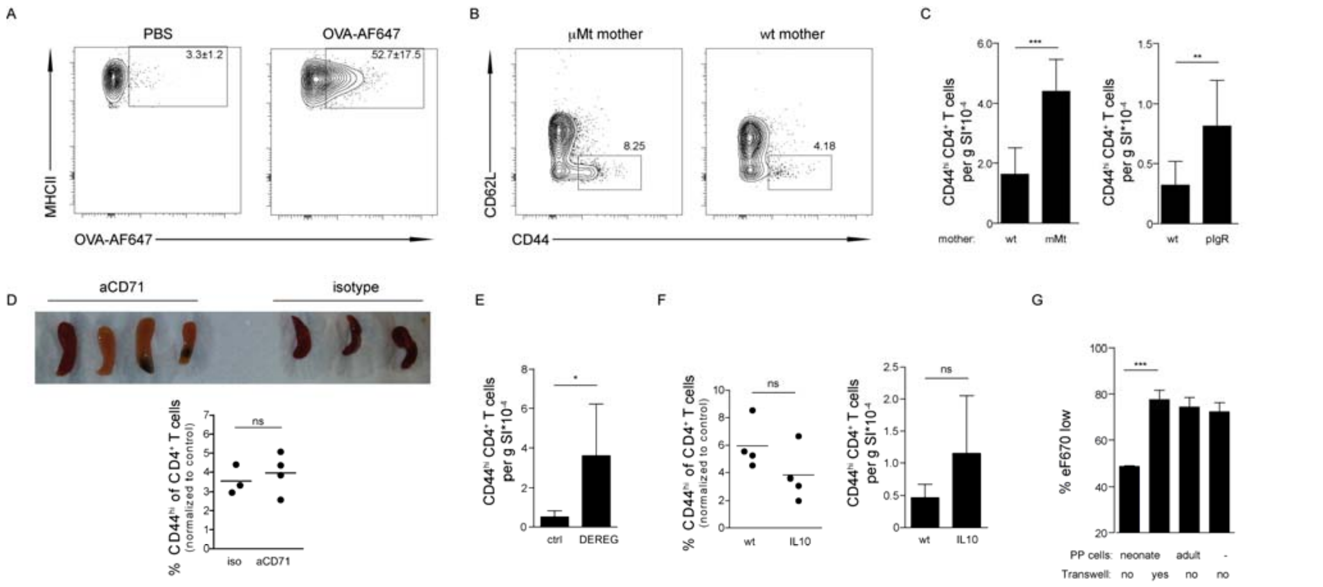

Supplementary Figure 4: Mechanisms that contribute to the suppression of neonatal small intestinal CD4 T lymphocyte maturation. (a) The capacity of intestinal myeloid APC to take up luminal antigen was measured by flow cytometric detection of AF647 in $CD11b^{+}MHCII^{+}$ cells isolated from the small intestine of 6-day-old neonatal mice 4 h after oral gavage of 20 μg OVA-AF647. (n=3, mean±SD). **(b)** Representative FACS plot of $CD44^{hi}CD62L^{-}$ CD4 T lymphocyte s in the Peyer's patches of 12-day-old μMt sufficient neonates fed by wt or μMt deficient dams (n=4). **(c)** Comparative analysis of absolute numbers of $CD44^{hi}$ CD4 T lymphocytes in the small intestine of 11-day-old B cell ($\mu MT^{+/-}$ or $\mu MT^{+/+}$) (left panel) and IgA sufficient ($pIgR^{+/-}$ or $pIgR^{+/+}$)(right panel) neonates fed by B cell or IgA sufficient (wt mother) or deficient ($\mu MT^{-/-}$ or $pIgR^{-/-}$ mother) dams. (n=2 litters from 2 experiments, mean± SD; unpaired Student's t test, ***, p<0.001, **, p<0.01). **(d)** Depletion assessment of $CD71^{+}$ cells in neonatal mice treated with antiCD71 antibody (upper panel) and comparative analysis of the percentage of $CD44^{hi}$ CD4 T lymphocytes in the small intestine of antiCD71 treated neonates at 9 days of age (lower panel). (n=3-4, mean, unpaired Student's t test; ns, not significant). **(e)** Absolute numbers of $CD44^{hi}$ cells among CD4 T lymphocytes normalized to tissue weight (using non transgenic littermate controls as a reference gate) in the SI of 11-day-old DEREG mice and non-transgenic littermate controls treated with diphtheria toxin on days 1/2/5/6 (n=3 litters, one representative of 3 experiments, mean± SD; unpaired Student's t test, *, p<0.05). **(f)** Percentage (left panel) and absolute numbers of $CD44^{hi}$ cells among CD4 T lymphocytes normalized to tissue weight (right panel) in the SI of 11-day-old $IL10^{-/-}$ and wt mice (n=2 litters, one representative of 2 experiments, mean± SD; unpaired Student's t test, ns, not significant). **(g)** Comparative proliferation assay culturing OVA-loaded BMDCs, eFluor670 labeled OTII T lymphocytes together with neonatal (11d) or adult PP cells for 3 days at a 4:1 ratio of PP cells to OTII cell respectively with or without a transwell (0.4 μm) separating both cell types. (n=3 technical replicates, representative of 3 similar independent experiments, mean; one-way ANOVA, Bonferroni's post test, ***, p<0.001).
